# Using logic model methods in systematic review synthesis: describing complex pathways in referral management interventions

**DOI:** 10.1186/1471-2288-14-62

**Published:** 2014-05-10

**Authors:** Susan K Baxter, Lindsay Blank, Helen Buckley Woods, Nick Payne, Melanie Rimmer, Elizabeth Goyder

**Affiliations:** 1 School of Health and Related Research, University of Sheffield, Regent Court, 30 Regent Street, Sheffield S14DA, UK

**Keywords:** Systematic review, Methodology, Evidence synthesis, Logic model, Demand management, Referral systems, Referral management

## Abstract

**Background:**

There is increasing interest in innovative methods to carry out systematic reviews of complex interventions. Theory-based approaches, such as logic models, have been suggested as a means of providing additional insights beyond that obtained via conventional review methods.

**Methods:**

This paper reports the use of an innovative method which combines systematic review processes with logic model techniques to synthesise a broad range of literature. The potential value of the model produced was explored with stakeholders.

**Results:**

The review identified 295 papers that met the inclusion criteria. The papers consisted of 141 intervention studies and 154 non-intervention quantitative and qualitative articles. A logic model was systematically built from these studies. The model outlines interventions, short term outcomes, moderating and mediating factors and long term demand management outcomes and impacts. Interventions were grouped into typologies of practitioner education, process change, system change, and patient intervention. Short-term outcomes identified that may result from these interventions were changed physician or patient knowledge, beliefs or attitudes and also interventions related to changed doctor-patient interaction. A range of factors which may influence whether these outcomes lead to long term change were detailed. Demand management outcomes and intended impacts included content of referral, rate of referral, and doctor or patient satisfaction.

**Conclusions:**

The logic model details evidence and assumptions underpinning the complex pathway from interventions to demand management impact. The method offers a useful addition to systematic review methodologies.

**Trial registration number:**

PROSPERO registration number: CRD42013004037.

## Background

Worldwide shifts in demographics and disease patterns, accompanied by changes in societal expectations are driving up treatment costs. As a result of this, several strategies have been developed to manage the referral of patients for specialist care. In the United Kingdom (UK) referrals from primary care to secondary services are made by General Practitioners (GPs), who may be termed Family Physicians or Primary Care Providers in other health systems. These physicians in the UK act as the gatekeeper for patient access to secondary care, and are responsible for deciding which patients require referral to specialist care. Similar models are found in health care services in Australia, Denmark and the Netherlands however, this process differs from systems in other countries such as France and the United States of America.

As demand outstrips resources in the UK, the volume and appropriateness of referrals from primary care to specialist services has become a key concern. The term “demand management” is used to describe methods which monitor, direct or regulate patient referrals within the healthcare system. Evaluation of these referral management interventions however presents challenges for systematic review methodologies. Target outcomes are diverse, encompassing for example both the reduction of referrals and enhancing the optimal timing of referrals. Also, the interventions are varied and may target primary care, specialist services, or administration or infrastructure (such as triaging processes and referral management centres) [1].

In systematic review methodology there is increasing recognition of the need to evaluate not only what works, but the theory of why and how an intervention works [2]. The evaluation of complex interventions such as referral management therefore requires methods which move beyond reductionist approaches, to those which examine wider factors including mechanisms of change [3-5].

A logic model is a summary diagram which maps out an intervention and conjectured links between the intervention and anticipated outcomes in order to develop a summarised theory of how a complex intervention works. Logic models seek to uncover the theories of change or logic underpinning pathways from interventions to outcomes [2]. The aim is to identify assumptions which underpin links between interventions, and the intended short and long term outcomes and broader impacts [6]. While logic models have been used for some time in programme evaluation, their potential to make a contribution to systematic review methodology has been recognised only more recently. Anderson et al. [7] discuss their use at many points in the systematic review process including scoping the review, guiding the searching and identification stages, and during interpretation of the results. Referral management entails moving from a system that reacts in an ad hoc way to increasing needs, to one which is able to plan, direct and optimise services in order to optimise demand, capacity and access across an area. Uncovering the assumptions and processes within a referral management intervention therefore requires an understanding of whole systems and assumptions, which a logic model methodology is well placed to address.

A number of benefits from using logic models have been proposed including: identification of different understandings or theories about how an intervention should work; clarification of which interventions lead to which outcomes; providing a summary of the key elements of an intervention; and the generation of testable hypotheses [8]. These advantages relate to the power of diagrammatic representation as a communication tool. Logic models have the potential to make systematic reviews “more transparent and more cogent” to decision-makers [7]. The use of alternative methods of synthesis and presentation of reviews is also worthy of consideration given the poor awareness and use of systematic review results amongst clinicians [9]. In addition, logic models may move systematic review findings beyond the oft-repeated conclusion that more evidence is needed [7].

While the potential benefit as a communication tool has been emphasised, there has been limited evaluation of logic models. In this study we aimed to further develop and evaluate the use of logic models as synthesis tools, during a systematic review of interventions to manage referrals from primary care to hospital specialists.

## Methods

The method we used built on previous work by members of the team [10,11]. The approach combines conventional rigorous and transparent review methods (systematic searching, identification, selection and extraction of papers for review, and appraisal of potential bias amongst included studies) with a logic model synthesis of data. The building of models systematically from the evidence contrasts to the approach typically adopted, whereby logic models are built by discussion and consensus at meetings of stakeholders or expert groups. The processes followed are described in further detail below.

### Search strategy

A study protocol was devised (PROSPERO registration number: CRD42013004037) to guide the review which outlined the research questions, search strategy, inclusion criteria, and methods to be used. The primary research question was “what can be learned from the international evidence on interventions to manage referral from primary to specialist care?” Secondary questions were “what factors affect the applicability of international evidence in the UK”, and “what are the pathways from interventions to improved outcomes?”

Systematic searches of published and unpublished (grey literature) sources from healthcare, and other industries were undertaken. Rather than a single search, an iterative (a number of different searches) and emergent approach (the understanding of the question develops throughout the process), was taken to identify evidence [12,13]. As the model was constructed, further searches were required in order to seek additional evidence where there were gaps in the chain of reasoning as described below. An audit table of the search process was kept, with date of search, search terms/strategy, database searched, number of hits, keywords and other comments included, in order that searches were transparent, systematic and replicable.

Searches took place between November 2012 and July 2013. A broad range of electronic databases was searched in order to reflect the diffuse nature of the evidence (see Additional file 1). Citation searches of included articles and other systematic reviews were also undertaken and relevant reviews articles were used to identify studies. Grey literature (in the form of published or unpublished reports, data published on websites, in government policy documents or in books) was searched for using OpenGrey, Greysource, and Google Scholar electronic databases. Hand searching of reference lists of all included articles was also undertaken; including relevant systematic reviews.

### Identification of studies

Inclusion/exclusion criteria were developed using the established PICO framework [14]. Participants included all primary care physicians, hospital specialists, and their patients. Interventions included were those which aimed to influence and/or affect referral from primary care to specialist services by having an impact on the referral practices of the primary physician. Studies using any comparator group were eligible for inclusion, and all outcomes relating to referral were considered. With the increasing recognition that a broad range of evidence is able to inform review findings, no restrictions were placed on study design with controlled, non-controlled (before and after) studies, as well as qualitative work examined. Studies eligible for inclusion were limited by date (January 2000 to July 2013). Articles in non-English languages with English abstracts were considered for translation (none were found to meet the inclusion criteria for the review). The key criterion for inclusion in the review was that a study was able to answer or inform the research questions.

### Selection of papers

Citations identified using the above search methods were imported into Reference Manager Version 12. The database was screened by two reviewers, with identification and coding of potential papers for inclusion. Full papers copies of potentially relevant articles were retrieved for further examination.

### Data extraction

A data extraction form was developed using the previous expertise of the review team, trialled using a small number of papers, and refined for use here. Data extractions were completed by one reviewer and checked by a second. Extracted data included: country of the study, study design, data collection method, aim of the study, detail of participants (number, any reported demographics), study methods/intervention details, comparator details if any, length of follow up, response and/or attrition rate, context (referral from what/who to what/who), outcome measures, main results, and reported associations.

### Quality appraisal

The potential for bias within each quantitative study was assessed drawing on work by the Cochrane Collaboration [15]. We slightly adapted their tool for assessing risk of bias in order that the appraisal would be suitable for our broader range of study designs. For the qualitative papers we adapted the Critical Appraisal Skills Checklist [16] to provide a similar format to the quantitative tool. In addition to assessing the quality of each individual paper we also considered the overall strength of evidence for papers grouped by typology, drawing on criteria used by Hoogendoom et al. [17]. Each group of papers was graded as providing either: stronger evidence (generally consistent findings in multiple higher quality studies); weaker evidence (generally consistent findings in one higher quality study and lower quality studies, or in multiple lower quality studies.); inconsistent evidence (<75% consistency findings in multiple studies) or very limited evidence (a single study). Strength of evidence appraisal was undertaken at a meeting of the research team to establish consensus.

### Logic model synthesis

Logic models typically adopt a left to right flow of “if....then” propositions to illustrate the chain of reasoning underpinning how interventions lead to immediate (or short term) outcomes and then to longer term outcomes and impacts. This lays out the logic or assumptions that underpin the pathway (in this case, what needs to happen in order for interventions with General Practitioners to impact on referral demand). In our approach, extracted data from the included papers across study designs are combined and treated as textual (qualitative) data. A process of charting, categorising and thematic synthesis [18] of the extracted quantitative intervention and qualitative data is used in order to identify individual elements of the model. A key part of the model is detailing the mechanism/s of change within the pathway and the moderating and mediating factors which may be associated with or influence outcomes [19] this is often referred to as the theory of change [2].

### Evaluation of the model

Following development of a draft model we sought feedback from stakeholders regarding the clarity of representation of the findings, and potential uses. We carried out group sessions with patient representatives, individual interviews and seminar presentations with GPs and consultants, and also interviews with commissioners (in the UK commissioning groups comprise individuals who are responsible for the process of planning, agreeing and monitoring services, having control of the budget to be spent). At these sessions we presented the draft model and asked for verbal comments regarding the clarity of the model as a way of understanding the review findings, any elements which seemed to be missing, elements which did not seem to make sense or fit participants knowledge or experience, and also how participants envisaged that the model could be used. We also gave out feedback forms for participants to provide written comments on these aspects. In addition to these sessions we circulated the draft model via email to topic experts for their input. The feedback we obtained was examined and discussed by the team in order to inform subsequent drafts of the model.

### Ethical approval

The main study was secondary research and therefore exempt from requiring ethical approval. Approval for the final feedback phase of the work was obtained from the University of Sheffield School of Health and Related Research ethics committee (reference 0599). Informed consent was obtained from all participants.

## Results

**Figure 1:**
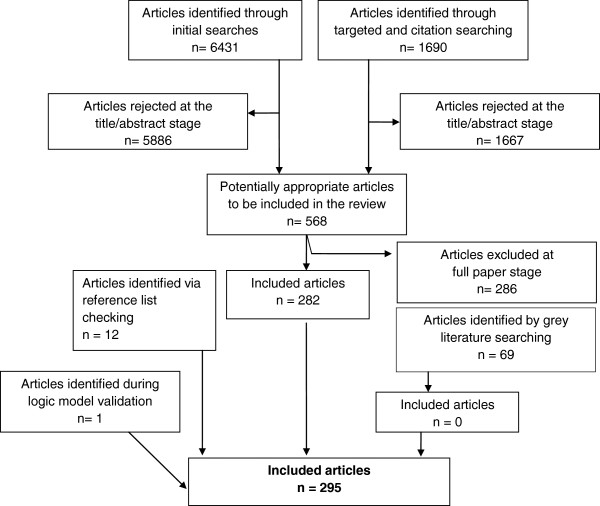
**The process of inclusion and exclusion.** A flow chart illustrating the process of paper identification.

The electronic searches generated a database of 8327 unique papers. Of these, 581 papers were selected for full paper review (see Additional file 1). After considering these and completing our further identification procedures, 295 papers were included in the review. Figure 1 illustrates the process of inclusion and exclusion. The included papers consisted of 141 intervention papers and 154 non-intervention papers. The 154 non-intervention papers included 33 qualitative studies and 121 quantitative studies (see Additional file 1).

**Figure 2:**
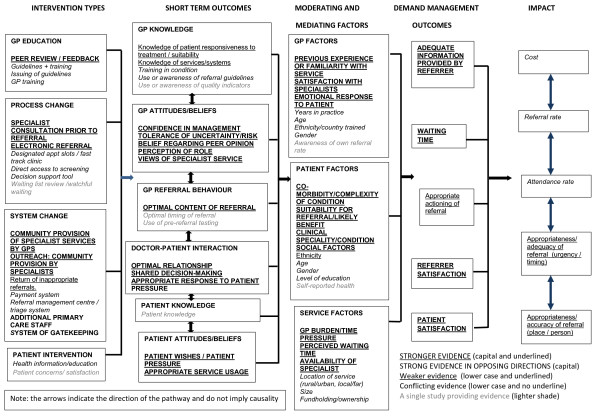
**The completed logic model built from examining the identified published literature.** The model illustrates the pathway between demand management interventions and intended impact. It reads from left to right, with a typology of demand management interventions in the first column, the immediate or short term outcomes following the interventions in the second column, then factors which may act as barriers to achievement of longer term outcomes in the mediating and moderating factors column, outcomes in terms of demand management at the level of physicians and their practice in the fourth column, and longer term system-wide impacts in the final column. The model indicates (via differing text types) where there was stronger or weaker evidence of links in the pathway.

A logic model was systematically developed from reviewing and synthesising these papers (see Figure 2). The model illustrates the elements in the pathway from demand management interventions to their intended impact. While this paper will refer to the emerging review findings which are currently undergoing peer review, its primary purpose is to describe and evaluate the methodology.

### Logic model development

Following data extraction and quality appraisal, the process of systematically constructing the logic model began. We developed the model column by column, underpinned by the evidence. The model contains five columns detailing the pathway from interventions to short-term outcomes; via moderating and mediating factors; to demand management outcomes; and finally demand management impact.

The first stage in building the model was to develop intervention typology tables from the extracted data, in order to begin the process of grouping and organising the intervention content and processes which would form the first column. This starting point in the pathway details the wide range of interventions which are reported in the literature. It groups these interventions into typologies of: practitioner education; process change; system change; and patient intervention. Within each of these boxes the specific types of interventions in each category have been listed, for example the GP education typology contains interventions targeting training sessions, peer feedback, and provision of guidelines. Process change interventions include electronic referral, direct access to screening and consultation with specialists prior to referral. System change interventions include additional staff in community, gate-keeping and payment systems. We found few examples of patient interventions. The model provides an indication of where the evidence is stronger or weaker. For example in regard to physician education it can be seen that peer review/feedback has stronger evidence underpinning its effectiveness, with the use of guidelines being underpinned by conflicting evidence of effectiveness. For all but two of the interventions, the evidence was for either none or some level of positive outcome on referral management. For the additional staff in primary care and the addition or removal of gatekeeping interventions however there was strong evidence that these could worsen referral management outcomes.

The interventions thus formed the starting point, and first column of the logic model. By developing a typology we were able to group and categorise the data, and begin to explore questions regarding which types of intervention may work, and what characteristics of interventions may be successful in managing patient referral.

The intervention studies used a wide range of outcomes to judge efficacy. A key aim of logic models is to uncover assumptions in the chain of reasoning between interventions and their expected impacts, and to develop a theory of change which sets out these implicit “if…then” pathways. The next stage in development of the model was therefore to begin to unpack these outcomes and assumptions regarding links between interventions and demand management impacts.

The outcomes were divided into those which were considered to be short-term outcomes, long term outcomes or result in broader impact on demand management systems. In order to do this we used “if…then” reasoning to deduce in what order outcomes needed to occur for these to then lead to the intended impact. Short-term outcomes were classified as those that impacted immediately or specifically on individual referrers, patients or referrals. Long term outcomes were categorised as those which had an effect more widely beyond the level of the individual GP, service or patient, and impact factors were those that would determine the effectiveness of referral management across whole health systems.

Outcomes and impacts reported in the intervention studies were identified and grouped by typology, by the stage in the pathway, and by the level of evidence. The outcomes column includes all those outcomes which were reported in the included papers. They encompass: whether or not the adequacy of information provided by the referrer to the specialist was improved; whether there was an improvement to patient waiting time; whether there was an increase in level of GP or patient satisfaction with services, and whether referrals were auctioned more appropriately. These outcomes form an important element of the pathway to the final impacts column and demonstrate the importance of identifying all the links in the chain of reasoning. For example the model outlines that referral information needs to be accurate in order that referrals may be directed to the most appropriate place or person. Interventions need to include evaluation of this interim outcome and not only consider impact measures such as rate of referral if they are to explore how and if an intervention is effective. Also, GP satisfaction with a service will determine where referrals are sent, and patient satisfaction may determine whether a costly appointment with a specialist is attended. Here again many studies we evaluated used only broad impact measures (such as referral rate) to evaluate outcomes rather than explore where the links in the pathway may be breaking down.

The impacts column contains all those impacts that were reported in the included literature. These were: the impact on referral rate/level; whether attendance rate increased; any impact on referrals being considered appropriate; any impact on the appropriateness of the timing of the referral; and the effect on healthcare cost. As can be seen from the model, the relationship between interventions and a wider impact on systems was challenging to demonstrate from the evidence.

Having developed the first and final two columns of the model, attention then turned to the key middle section. This phase of the work required detailed exploration of the change pathway to explore exactly how the interventions would act on participants in order to produce the demand management outcomes and impacts. The second and third columns of the model are core elements of the theory of change within the model.

While a small amount of data for these elements came from the intervention studies, the majority came from analysis and synthesis of the qualitative papers and non-intervention studies. Much of the intervention literature seemed to have a “black box” between the intervention and the long term impacts. This was a key area of the work where we employed iterative additional searching in order to seek evidence for associations, to ensure that the chain of reasoning was complete. For example, the first additional search aimed to explore evidence underpinning the assumption that increasing GP knowledge would lead to improved referral practice. The second additional search aimed to identify evidence underpinning the link between changes in referral systems and changed physician attitudes or behaviour. The search also sought evidence regarding specific outcomes following interventions to change patient knowledge, attitudes or behaviour.

The second column of the model details the short-term outcomes for individual GPs, patients, and GP services that may result from interventions. These are the factors which need to be changed within the referrer or referral, in order that the longer term outcomes and impacts will happen. The short-term outcomes we identified were: physician knowledge; physician beliefs/attitudes; physician behaviour; doctor-patient interaction; patient knowledge, or patient attitudes/beliefs or behaviour. Of note is the weaker evidence of physician knowledge change impacting on referrals, and greater evidence of change to physician attitudes and beliefs, and also doctor-patient interaction having an impact.

The third column (and final element to be completed) is another key part of the theory of change. This section identifies a range of factors which may be associated with or influence whether the short-term outcomes will lead to the intended longer term outcomes and impacts. This column examines the moderating and mediating variables which may act as predictors of whether an intervention will be successful. They can be considered as similar to the barriers and facilitators often described in qualitative studies. The model details a wide range of these moderating and mediating factors relating to: the physician; the patient; and the organisation. Of particular interest here is the conflicting evidence relating to physician and patient demographic factors (the subject of a large number of studies) influencing referral patterns, and the clearer picture regarding the influence of patient clinical and social factors in the referral process.

Having outlined the content of each column, the following provides an example of the flow of reasoning for one particular type of intervention underpinned by elements of the model. Much work in the UK has been directed towards issuing guidelines for GPs regarding who and when to refer, with the assumption that changed knowledge will lead to changed referral practice. However, the model questions these assumptions by indicating that there is conflicting evidence regarding the efficacy of this type of intervention, and also suggesting that there is weak evidence of interventions such as these leading to enhanced knowledge outcomes, Perhaps if the guidelines focused more on elements of the model where evidence is stronger such as addressing GP attitudes and beliefs (for example tolerance of risk) or behaviour (such as the optimal content of referral information) this may lead to more successful immediate outcomes. The model highlights however that the effect of any guideline intervention will also be modified by GP, patient and service factors, for example the complexity of the case, the GP”s emotional response to the patient and GP time pressure. These potential barriers need to be considered in the implementation of guideline interventions. If these elements can be addressed however, use of guidelines by GPs may enhance referrer or patient satisfaction, improve waiting time, or change the content of a referral and thus have a resulting impact at a service wide level.

### Evaluation of the model

Following development of the model we sought feedback from stakeholders regarding the clarity of representation of the findings, and potential uses. This consultation was carried out via individual and group discussion with practitioners, patient and the public representatives, commissioners (individuals who have responsibility for purchasing services), and by circulating the model to experts in the field. In total we received input from 44 individuals (15 GPs, five commissioners, seven patient and public representatives, and 17 hospital specialists from a range of clinical areas). Thirty eight of the respondents reported that they clearly understood the model however, four specialists described the model as overly complex and 2 patient representatives reported some confusion understanding it.

GPs in particular gave positive feedback, highlighting that it was a good fit with their experience of the way referrals are managed, and that it successfully conveyed the complexity of general practice. The model was also described positively as identifying the role of both the GPs’ and the patients’ attitudes and beliefs, and the doctor-patient interaction. Also, GPs noted with satisfaction that the model included the physicians’ emotional response to the patient, which resonated with their experiences. Most specialists also reported that the model was a good fit with their experience of factors influencing referral management. Potential uses of the model described were: as a tool for GP trainees and educators; as a teaching aid for undergraduate medical students; for analysing the demand management pathway when commissioning; for comparing what was being commissioned with what was evidence based; and to direct research into poorly evidenced areas.

Some of the feedback from participants concerned factors that had not been identified in the literature. For example the potential role of carers as well as the patient in doctor-patient interactions was highlighted, and the potential influence of being a GP temporarily covering a colleagues’ work. Some amendments were made to the model following this feedback, principally clarifying where there was no evidence versus inconclusive evidence, and editing terminology.

## Discussion

While referral management is often considered to have only a capacity-limiting function, the model was able to identify the true complexity of what is aiming to be achieved. Our model has added to the existing literature by setting out the chain of reasoning that underpins how and if interventions are to lead to their intended impacts, and made explicit the assumptions that underpin the process. The logic model is able to summarise a wealth of information regarding the findings of a systematic review on a single page. The visual presentation of this information was clearly understood by almost all professionals and all commissioners in our sample. This study therefore supports the value of logic models as communication tools. The effectiveness of the model for communicating findings to patients and the public however warrants further exploration. While four of the seven patient representatives in our group found value in the model, three found it lacked relevance to patients. While this was a very small sample, it would be worth exploring in the future whether the topic of the model contributed to this perception. Perhaps a model relating to a specific clinical condition rather than service delivery may be perceived of greater relevance to patients and the public.

The use of stakeholders in developing a theory of change has been recommended by other authors [20]. Participants in our sample were able to provide valuable input by suggesting areas where there were seeming gaps in evidence. Our method of building the model from the literature has sought to be systematic and evidence-based, rather than be influenced by expert/stakeholder opinion (as is more typically the process of logic model development). However, while it is important to be alert to potential sources of bias in the review process, it seems that the involvement of stakeholders for determining potential gaps in evidence alongside systematic identification processes should not be ignored. The logic model we have produced outlines only where we identified literature, and does not include the two areas suggested by the stakeholders. We debated whether these suggested areas should be added to the model, and concluded that it would be counter-intuitive in a model presenting the evidence to show areas of no evidence. It is possible that future development of the methodology could consider including “ghost boxes” or similar to indicate where experts or practitioners believe that there are links, however there is no current research to substantiate this.

We endeavoured to enhance the communicative power of the model by adopting a system of evaluating the strength of evidence underpinning elements. The determination of strength of evidence is a challenging area, with our adopted system likely to be the subject of debate. Other widely used methods of appraising the quality of evidence (such as that used by the Cochrane Collaboration [15]) typically use different checklists for different study designs. The method that we adopted was able to be inclusive of the diversity of types of evidence in our review. In selecting an approach we also aimed to move beyond a simple count of papers. This “more equals stronger” approach may be misleading as a greater number may be only an indicator of where work has been carried out, or is perhaps where a topic is more amenable to investigation. The evaluation we utilised included elements of both quantity and quality, together with the consideration of consistency. However, the volume of studies in the rating was still influential. While we believe that the strength of evidence indicator adds considerably to the model and review findings, we recognise that there is still work to be done in refining this aspect of the method.

Our process of synthesising the data to develop the logic model draws on methodological developments in the area of qualitative evidence synthesis [18,21]. Our use of categorising and charting to build elements also draws on techniques of Framework Analysis [22] which is commonly used as a method of qualitative data analysis in policy research. The Framework Method may be particularly useful to underpin this process as it is highly systematic method of categorizing and organizing data [23]. By its inclusion of a diverse range of evidence, our method also resonates with the growing use of mixed-methods research which appreciates the contribution of both qualitative and quantitative evidence to answering a research question. While our method utilises the model for synthesis at the latter end of a systematic review, logic models have been suggested as being of value at various stages of the process [7]. Recently it has been proposed that logic models should be added to the established PICOs framework for establishing review parameters [24] in the initial stages.

In order to be of value, a visual representation should stand up to scrutiny so that concepts and meaning can be grasped by others and stimulate discussion [21]. We believe that evaluation of our model indicates that it met these requirements. The use of diagrams to explain complex interventions has been criticised in the past on the grounds that it can fail to identify mechanisms of change [19]. We argue that by using a wide range of literature and employing methods of iterative searching to examine potential associations, that this potential limitation can be overcome. Vogel [25] emphasised that diagrams should combine simplicity with validity – an acknowledgement of complexity but recognition that things are more complex than can be described. The vast majority of feedback on the model reported that this complexity represented the area as they knew it, and that this was a key asset of the model.

## Conclusions

This work has demonstrated the potential value of a logic model synthesis approach to systematic review methodologies. In particular for this piece of work, the method proved valuable in unpicking the complexity of the area, illuminating multiple outcomes and potential impacts, and highlighting a range of factors that need to be considered if interventions are to lead to intended impacts.

## Competing interests

The authors declare that they have no competing interests.

## Authors’ contribution

SB was a reviewer and led development of the logic model. LB was principal investigator and lead reviewer. HB carried out the searches. NP and EG provided methodological and topic expertise throughout the work. MR carried out the evaluation phase. All members of the team read and commented on drafts of this paper.

## Pre-publication history

The pre-publication history for this paper can be accessed here:

http://www.biomedcentral.com/1471-2288/14/62/prepub

## Supplementary Material

Additional file 1 Using logic model methods in systematic review synthesis: describing complex pathways in referral management interventions. Click here for file
