# Supplementary material for: Using logic model methods in systematic review synthesis: describing complex pathways in referral management interventions
Source: BMC Med Res Methodol. 2014 May 10;14:62. doi: 10.1186/1471-2288-14-62 (PMC4028001; doi:10.1186/1471-2288-14-62)
Supplement: Additional file 1 — Using logic model methods in systematic review synthesis: describing complex pathways in referral management interventions. [file 1471-2288-14-62-S1.docx]

**Additional file 1**

**Using logic model methods in systematic review synthesis: describing complex pathways in referral management interventions.**

**Additional online material**

**Databases searched**

MEDLINE In-Process & Other Non-Indexed Citations and MEDLINE via Ovid <1946 to Present>

Cochrane Database of Systematic Reviews

Cochrane Central Register of Controlled Trials

Cochrane Methodology Register

Database of Abstracts of Reviews of Effects

Health Technology Assessment Database

NHS Economic Evaluation Database

All accessed via the Cochrane Library, published by John Wiley and Sons Ltd <from inception>

CINAHL via EBSCO <from inception>

Embase via Ovid <1974 to 2012 November 13>

PsycINFO via Ovid <1806 to November Week 1 2012>

Science Citation Index, Social Science Citation Index, Science Conference Papers Index, Social Science Conference Papers Index via Web of Science published by Thomson Reuters <from inception>

Scopus via Elsevier <from inception>

Applied Social Sciences Index and Abstracts (ASSIA) via ProQuest <from inception>

Sociological abstracts via ProQuest <from inception>

Social policy and practice via Ovid < 1890’s to October 2012>

International Bibliography of the Social Sciences (IBSS) via Proquest <from inception>

HMIC via NHS Evidence <from inception>

Health business Elite via EBSCO <from inception>

Business source premier via EBSCO <from inception>

Emerald management reviews via http://www.emeraldinsight.com/products/reviews/index.htm <from inception>

EPPI Centre databases: Bibliomap, Database of Promoting Health Effectiveness Reviews (DoPHER), Trials Register of Promoting Health Interventions (TRoPHI) via http://eppi.ioe.ac.uk/ <from inception>

OpenGrey via http://www.opengrey.eu/ <from inception>

Opensource via http://www.greynet.org/greysourceindex.html <from inception>

Google Scholar via http://scholar.google.co.uk/ <from inception>

**Sample search strategies**

**Initial search**

Database: Ovid MEDLINE(R) In-Process & Other Non-Indexed Citations and Ovid MEDLINE(R) <1946 to Present>

Search Strategy:

1 *Primary Health Care/ (31226)

2 (primary care or general practitioner$ or gp).ti. (38162)

3 *Family practice/ or *General practitioners/ (38225)

4 1 or 2 or 3 (83924)

5 (referral or referred or refer).ti. (10316)

6 demand management.ti,ab. (141)

7 *"Referral and Consultation"/ (17682)

8 Specialization/ (20898)

9 5 or 6 or 7 or 8 (43885)

10 4 and 9 (4328)

11 limit 10 to yr="2000 -Current" (1978)

**Phrase search**

Database: Ovid MEDLINE(R) In-Process & Other Non-Indexed Citations and Ovid MEDLINE(R) <1946 to Present>

Search Strategy:

1 "referral management centre$".mp. [mp=title, abstract, original title, name of substance word, subject heading word, keyword heading word, protocol supplementary concept, rare disease supplementary concept, unique identifier] (7)

2 limit 1 to yr="2000 -Current" (7)

**“Clinical reasoning” search**

Database: Ovid MEDLINE(R) In-Process & Other Non-Indexed Citations and Ovid MEDLINE(R) <1946 to Present>

Search Strategy:

1 (evidence based adj (practice or medicine)).ti,ab. (12476)

2 Evidence-Based Practice/ or Evidence-Based Medicine/ (52802)

3 1 or 2 (57837)

4 (gp or general practitioner).ti,ab. (36865)

5 General Practice/ or Family Practice/ or General Practitioners/ (62491)

6 4 or 5 (90428)

7 (clinical reasoning or clinical judgement or problem solving or decision making or critical thinking).ti,ab. (73412)

8 Clinical Competence/ (60196)

9 Problem Solving/ (20285)

10 7 or 8 or 9 (145906)

11 3 and 6 and 10 (170)

**“Systems” search**

Database: Ovid MEDLINE(R) In-Process & Other Non-Indexed Citations and Ovid MEDLINE(R) <1946 to Present>

Search Strategy:

1 referral.ti,ab. (54436)

2 exp "Referral and Consultation"/ (54186)

3 1 or 2 (96206)

4 *"Appointments and Schedules"/ (3226)

5 *Computer Systems/ (3932)

6 *Information Systems/ (11904)

7 *Physician Incentive Plans/ (1225)

8 (Proforma$ or checklist$ or appointment$ or direct access or IT system* or informatics or software or electronic or outreach clinic$ or specialist nurse$ or patient review$ or gatekeep$ or nominated destination or financial incentive$ or payment$).ti,ab. (233486)

9 4 or 5 or 6 or 7 or 8 (249981)

10 3 and 9 (5345)

11 ((doctor$ or gp$ or general practitioner$ or physician$) adj3 (attitude$ or behavio?r or knowledge)).ti,ab. (9324)

12 (patient$ adj3 (attitude$ or behavio?r or knowledge)).ti,ab. (19104)

13 exp *attitude to health/ or *health knowledge, attitudes, practice/ (136183)

14 11 or 12 or 13 (158409)

15 10 and 14 (574)

**Table of included studies**

| Aache et al. 2011 | Cross sectional |
| --- | --- |
| White 2000 | Survey |
| Abel and Thompson 2011 | Qualitative |
| Adams 2012 | Before and after |
| Ahluwalia et al. 2009 | Survey |
| Akbari 2012 | Before and after |
| Albertson 2002 | Sequential prospective before and after study |
| Albertson et al. 2000 | Survey |
| Alexander & Fraser 2008 | Survey |
| Allareddy 2007 | Qualitative |
| Angstman 2009 | Survey |
| Anthony et al. 2010 | Mixed methods |
| Ashworth et al. 2002 | Patient record analysis |
| Baker et al. 2006 | Qualitative |
| Balduf 2008 | Survey |
| Banait et al. 2003 | Cluster RCT |
| Barnett et al. 2011 | Survey |
| Bederman 2010 | Delphi consensus |
| Beel 2008 | Qualitative |
| Bekkelund 2001 | Survey |
| Belgamwar 2012 | Patient record analysis |
| Bennett 2001 | Cluster RCT |
| Berendsen 2007 | Qualitative |
| Berendsen et al. 2010 | Survey |
| Bertakis 2001 | Patient record analysis |
| Bertakis et al. 2001 | Mixed methods |
| Bhalla 2002 | Case control |
| Blundell 2011 | Survey |
| Blundell 2012 | Qualitative |
| Bolanos-Carmona et al. 2002 | Patient record analysis |
| Boulware 2006 | Questionnaire |
| Bowling 2000 | Survey |
| Bowling 2006 | Qualitative |
| Bridgman et al. 2005 | Controlled before and after |
| Brien et al. 2008 | Qualitative |
| Bruynincksx et al. 2009 | Patient record analysis |
| Bruyninckx et al. 2009 | Qualitative |
| Burns 2000 | Patient record analysis |
| Callaway & Frisch, 2000 | Service data review |
| Calnan et al. 2007 | Qualitative |
| Campbell et al. 2003 | Cluster RCT |
| Carlsen et al. 2008 | Survey |
| Chan 2003 | Physician claims database analysis |
| Chauhan 2012 | Patient record analysis |
| Chen 2010 | Before and after |
| Chen et al. 2005 | Patient record analysis |
| Chew-Graham et al. 2008 | Qualitative |
| Chung et al. 2010 | Patient record analysis |
| Clarke 2010 | Survey |
| Clemence 2003 | Qualitative |
| Cohen et al. 2013 | Patient record analysis |
| Cooper 2012 | Evaluation/audit report |
| Cooper and Wojnarowska 2001 | Patient record analysis |
| Cornford 2004 | Qualitative |
| Coulston et al. 2008 | Survey |
| Cox 2013 | Before and after |
| Coyle et al. 2011 | Survey |
| Cusack & Buckley 2005 | Before and after |
| Dagneaux 2012 | Qualitative |
| Dale & Goodsman 2000 | Survey |
| Damask trial team, 2008 | Cost effectiveness analysis alongside RCT |
| Davies 2006 | Mixed methods |
| Dearman et al. 2006 | Patient record analysis |
| Delnoij et al. 2000 | analysis of OECD data |
| Delva et al. 2011 | Survey |
| Dennison et al. 2006 | Cross sectional evaluation.gastroenterology clinics. |
| Dey 2004 | Cluster RCT |
| Dhillon et al. 2003 | RCT |
| Dodds 2004 | Survey |
| Donohoe 2000 | Cluster RCT |
| Eccles 2001 | RCT |
| Edwards 2001 | Qualitative |
| Eley 2010 | Audit |
| Elhayany et al. 2000 | Patient record analysis |
| Ellard 2012 | Before and after with control |
| Elwyn et al. 2007 | Before and after |
| Emery 2007 | Cluster RCT |
| Eminovic 2009 | Cluster RCT |
| Emmerson 2003 | Evaluation questionnaire |
| Engers 2005 | Cluster RCT |
| Espeland 2003 | Qualitative |
| Evans 2009 | Evaluation |
| Evans 2011 | Service development project |
| Fearn 2009 | Before and after |
| Feeney 2007 | Patient record analysis |
| Felker et al. 2004 | Before and after |
| Ferris 2002 | Before and after |
| Ferris 2002 | Before and after |
| Ferris et al. 2001 | Before and after |
| Ferriter 2006 | Before and after |
| Forest 2003 | Patient record analysis |
| Forrest 2007 | Survey |
| Forrest et al. 2002 | Survey |
| Forrest et al. 2006 | Survey |
| Franks et al. 2000 | Mixed methods |
| Franz 2010 | Qualitative |
| Freed et al. 2003 | Survey |
| Fucito 2003 | Survey |
| Gandhi 2000 | Survey |
| Gandhi 2008 | Non-randomised controlled trial |
| Glaves 2005 | Before and after |
| Glozier et al. 2007 | Mixed methods |
| Gough-Palmer et al. 2009 | Retrospective record analysis |
| Grace 2008 | Survey |
| Green et al. 2008 | Survey |
| Greenaway & Fortune 2006 | Mixed methods |
| Greer et al. 2011 | Survey |
| Greiver 2005 | Cluster RCT |
| Griffiths 2006 | Cluster RCT |
| Gross 2000 | Survey |
| Gruen et al. 2002 | Survey |
| Guevara et al. 2009 | Survey |
| Gurden et al. 2012 | Before and after |
| Hands 2001 | Before and after |
| Harlan 2006 | Survey |
| Harrington et al. 2001 | Case series |
| Harris 2011 | Survey |
| Harris et al. 2011 | Mixed methods |
| Harvey et al. 2005 | Survey |
| Heaney 2001 | RCT |
| Hemingway et al. 2006 | Before and after |
| Hermush et al. 2009 | Before and after. |
| Hill 2000 | Before and after |
| Hilty 2006 | Before and after |
| Hockney 2004 | longitudinal (no control) evaluation |
| Holley 2010 | Qualitative |
| Hughes-Anderson 2002 | Before and after |
| Hugo et al. 2000 | Patient record analysis |
| Hyman 2001 | Survey |
| Idiculla 2000 | retrospective analysis of referral letters |
| Imkampe 2006 | Before and after |
| Iversen & Luras 2000 | Economic analysis and modelling |
| Jaatinen 2002 | RCT |
| Jiwa 2004b | Non-randomised controlled trial |
| Jiwa 2006 | Cluster RCT |
| Jiwa 2012 | Before and after |
| Jiwa et al. 2008 | Survey |
| Jiwa et al. 2009 | Patient record analysis |
| Johnson 2011 | Qualitative |
| Johnson et al. 2008 | Survey |
| Johnson et al. 2008 | Cross sectional survey |
| Johnson et al. 2011 | Survey |
| Jorgensen et al. 2001 | Mixed methods |
| Joyce 2000 | Retrospective study |
| Julian 2007 | Non-randomised controlled trial |
| Junghams 2007 | RCT |
| Kazje 2004 | Survey |
| Kennedy et al. 2012 | Retrospective audit |
| Kerry 2000 | RCT |
| Khan 2008 | Cohort study |
| Kier et al. 2012 | Survey |
| Kim 2004 | Survey |
| Kim 2009 | Survey |
| Kim 2009 | Survey |
| Kim-Hwang 2010 | Survey |
| Kinchen 2004 | Survey |
| King 2001 | Before and after |
| Kisely 2002 | Mixed methods |
| Knab 2001 | Before and after |
| Knight 2003 | Qualitative |
| Knol 2006 | Before and after |
| Kousgaard 2003 | RCT |
| Kvaerner 2007 | Survey |
| Lakha et al. 2011 | Survey |
| Lam 2011 | Survey |
| Lambert 2001 | Survey |
| Leggett 2004 | RCT |
| Leiba 2002 | Non-randomised controlled trial |
| Lester 2009 | Cluster RCT |
| Levell 2011 | Before and after |
| Lewis 2000 | Qualitative |
| Little et al. 2004 | Survey |
| Love 2005 | Patient record analysis |
| Lucassen 2001 | Before and after |
| Maddison 2004 | Before and after |
| Magill et al. 2009 | Before and after |
| Malcolm 2008 | Survey |
| Malik 2007 | Audit of patient data |
| Mariotti 2008 | Audit. |
| Massey 2004 | Survey |
| Matowe et al. 2002 | Before and after |
| McBride et al. 2010 | Patient record analysis |
| McGarry 2009 | Survey |
| McGowan 2008 | RCT |
| McKenna et al. 2005 | Survey |
| McKoy 2004 | Before and after |
| McNally et al. 2003 | Before and after |
| Melia et al. 2008 | Before and after |
| Mitchell 2012 | Mixed methods |
| Montgomery et al. 2006 | Qualitative |
| Moore et al. 2000 | Survey |
| Morgan 2007 | Qualitative |
| Morrison et al. 2001 | RCT |
| Morsi et al. 2012 | Survey |
| Mulvaney 2005 | Survey |
| Musila 2011 | Guideline development group |
| Naccarella et al. 2008 | Survey |
| Nandy et al. 2001 | Qualitative |
| Navaneethan et al. 2010 | Mixed methods |
| Nicholson et al. 2006 | Survey |
| Nicholson et al. 2006 | Service evaluation |
| Nielsen 2003 | RCT |
| O Sullivan et al. 2005 | Patient record analysis |
| O’Byrne et al. 2010 | Patient record analysis |
| O’Neill 2005 | Survey |
| Olson et al. 2012 | Survey |
| Patterson 2004 | Cohort study |
| Pfeiffer et al. 2011 | Patient record analysis |
| Pfeiffer et al. 2011 | Retrospective analysis of clinic data |
| Philichi & Yuwono 2010 | Survey |
| Pomeroy and Cant 2010 | Mixed methods |
| Potter 2007 | Prospective cohort study. |
| Prades et al. 2011 | Mixed methods |
| Pryor and Knowles 2001 | Survey |
| Ramathan et al. 2011 | Survey |
| Ramchandiani 2002 | Survey |
| Ramsay et al. 2003 | Follow up to RCT. |
| Ridsdale 2007 | Patient record analysis |
| Ridsdale 2009 | Survey |
| Ringard 2010 | Survey |
| Robinson and Taylor 2010 | Patient record analysis |
| Robling et al. 2002 | RCT |
| Rose et al. 2001 | Survey |
| Rosemann 2006 | Survey |
| Rosen [Kings Fund Report] 2007 | Qualitative |
| Rosen 2006 | observational comparative cohort design |
| Rowlands 2001 | Qualitative |
| Rowlands et al. 2003 | Questionnaire data collected during RCT |
| Rushton et al. 2002 | Survey |
| Ruston, 2004 | Qualitative |
| Salisbury 2006 | RCT |
| Samant 2007 | Survey |
| Sanderson 2002 | RCT |
| Sauro 2004 | Non-randomised controlled trial |
| Scheerers 2007 | Survey |
| Schillinger et al. 2000 | RCT |
| Schulpen et al. 2003 | Non-randomised controlled trial |
| Shadd et al. 2011 | Patient record analysis |
| Shariff 2010 | Cohort study |
| Shaw 2006 | Cluster RCT |
| Sigel and Leiper 2004 | Qualitative |
| Simpson 2003 | Before and after |
| Simpson et al. 2010 | Mixed methods |
| Slade et al. 2008 | Cluster RCT |
| Soerensen 2009 | Patient record analysis |
| Spatagpra et al. 2005 | Before and after |
| Stainkey et al. 2010 | Service evaluation |
| Standing 2001 | Before and after |
| Stavrou et al. 2009 | Qualitative |
| Steele 2010 | Survey |
| Steele et al. 2012 | Survey |
| Stoves 2010 | Before and after |
| Suris et al. 2007 | Before and after |
| Sved-Williams 2010 | Before and after |
| Swarzrauber 2002 | Survey |
| Tadros 2009 | RCT |
| Taggarshe 2006 | Mixed methods |
| Tan et al. 2006 | Referral pattern analysis |
| Thomas 2003 | Cluster RCT |
| Thomas et al. 2010 | Analysis of referral data. |
| Thorsen 2012 | Qualitative |
| Tierney 2003 | RCT |
| Todman et al. 2011 | Survey |
| Townsley et al. 2003 | Survey |
| Trude et al. 2003 | Survey |
| Tucker 2003 | Mixed methods |
| Twomey 2003 | Before and after |
| Tzaribachev 2009 | Patient record analysis |
| van Bokhoven et al. 2012 | Cluster RCT |
| Van der Weijden et al. 2002 | Qualitative |
| Van Dijk 2013 | Before and after |
| Van Dijk et al. 2010 | Retrospective patient data analysis. |
| Van Dijk et al. 2011 | Retrospective medical record analysis |
| Vardy et al, 2008 | Mixed methods |
| Vinker et al. 2007 | Mixed methods |
| Vlek et al. 2003 | RCT |
| Vulto et al. 2009 | Survey |
| Wakefield et al. 2012 | Survey |
| Walders 2003 | Survey |
| Walkowski 2007 | Cluster RCT |
| Wallace 2004 | RCT |
| Wassenaar et al. 2007 | Survey |
| Watson 2001 | Survey |
| Watson 2001 | Cluster RCT |
| Watson 2005 | retrospective case note review |
| West 2007 | Before and after |
| White 2004 | Audit of referral letters |
| Whited 2002 | RCT |
| Whited 2004 | survey |
| Whitting 2011 | Observational |
| Wilkes et al. 2009 | Qualitative |
| Wilson 2006 | Cluster RCT |
| Wolters 2005 | RCT |
| Wong et al. 2000 | Controlled before and after |
| Wright et al. 2006 | Controlled before and after |
| Wylie 2001 | Survey |
| Xu et al. 2002 | Patient record analysis |
| Young et al. 2010 | Qualitative |
| Zielinski et al. 2008 | Patient record analysis |
